# Robust Physiological Metrics From Sparsely Sampled Networks

**DOI:** 10.3389/fphys.2021.624097

**Published:** 2021-02-10

**Authors:** Alan A. Cohen, Sebastien Leblanc, Xavier Roucou

**Affiliations:** ^1^Groupe de Recherche PRIMUS, Département de Médecine de Famille et de Médecine d’Urgence, Université de Sherbrooke, Sherbrooke, QC, Canada; ^2^Centre de Recherche, Centre Hospitalier Universitaire de Sherbrooke (CRCHUS), Sherbrooke, QC, Canada; ^3^Research Center on Aging, CIUSSS-de-l’Estrie-CHUS, Sherbrooke, QC, Canada; ^4^Département de Biochimie et de Génomique Fonctionnelle, Université de Sherbrooke, Sherbrooke, QC, Canada

**Keywords:** network physiology, complex dynamic system, big data, alternative proteins, systems biology, statistical distance

## Abstract

Physiological and biochemical networks are highly complex, involving thousands of nodes as well as a hierarchical structure. True network structure is also rarely known. This presents major challenges for applying classical network theory to these networks. However, complex systems generally share the property of having a diffuse or distributed signal. Accordingly, we should predict that system state can be robustly estimated with sparse sampling, and with limited knowledge of true network structure. In this review, we summarize recent findings from several methodologies to estimate system state via a limited sample of biomarkers, notably Mahalanobis distance, principal components analysis, and cluster analysis. While statistically simple, these methods allow novel characterizations of system state when applied judiciously. Broadly, system state can often be estimated even from random samples of biomarkers. Furthermore, appropriate methods can detect emergent underlying physiological structure from this sparse data. We propose that approaches such as these are a powerful tool to understand physiology, and could lead to a new understanding and mapping of the functional implications of biological variation.

## Introduction

Complex systems theorists have long viewed biological networks as one of the prime examples of complex systems (Holland, 1992; Mobus and Kalton, 2014), but biologists themselves, with some notable exceptions (e.g., Kitano, 2002; Tieri et al., 2010), have often been more reticent, preferring to understand biological signaling pathways from a more linear and reductionist perspective. This is starting to change, with systems biology gradually moving from simple inventories of large numbers of molecules to network-based approaches [e.g., Ingenuity Pathway Analysis, Kyoto Encyclopedia of Genes and Genomes (KEGG)-pathways, gene ontology (GO)-terms (Kanehisa and Goto, 2000; Harris et al., 2004; Krämer et al., 2014)]. Indeed, this entire Research Topic, one of the largest in the history of Frontiers Research Topics, is devoted to Network Physiology.

Indeed, drawing on insights from classical network theory (Albert and Barabási, 2002), over the last ∼10 years, network physiology, and the related field of network medicine, have been making great strides in bringing network thinking into the biomedical realm [Network medicine tends to focus more on genetic and molecular networks (Barabási et al., 2011), while network physiology focuses more on the temporal coordination of physiological function across systems, often at higher levels of organization, such as organs and organ systems (Ivanov et al., 2016), though the distinction is not always clear]. These methods are contributing numerous insights, such as coordination of sleep cycles across brain waves and muscle groups (Bashan et al., 2012) or understanding motor neuron function in *Caenorhabditis elegans* (Yan et al., 2017). Perhaps one of the most important insights is simply the repeated validation that there is coordination across biological networks, and thus that networks must be studied as ensembles, not as pieces. For example, there is now clear evidence that temporal dynamics of organ function show clear coordination across organ systems (Bartsch et al., 2015). Accompanying such insights come methods to quantify these network dynamics, such as time delay stability (TDS) and accompanying graphical methods (Bashan et al., 2012; Bartsch et al., 2015). It doesn’t require much thinking about the principles of evolution, optimization, and organismal function to understand why this is expected; the surprise is that physiology existed for so long without looking for this coordination, or without considering the higher-order perspective that it implies. For the purposes of this review, an additional key insight is that this coordination leads to a limited number of largely discrete states of the larger system, with multiple subsystems changing together in abrupt and coordinated fashion (Bashan et al., 2012; Bartsch et al., 2014, 2015). For example, multiple organ systems change together at transitions between sleep stages (Bartsch et al., 2015). Similar principles apply at other hierarchical levels of biology, for example in β-cell regulation in response to glucose (Podobnik et al., 2020). This leads to the important conclusion that there are *biological attractor states*, discrete states toward which physiology/biology converges, and between which it shifts.

Nonetheless, there are still major challenges in applying classical network theory to many aspects of biological networks. Classical network theory is based on the ability to map networks relatively exhaustively in order to estimate properties such as connectedness, modularity, etc. (Strogatz, 2001; Barabási, 2016), but biological networks imply multiple levels of organization, interactions of different types of structure (physical, informational, etc.), and networks that, on the molecular level, are still very poorly mapped. Additional approaches are thus needed for when our sparse understanding of network structure limits the applications of classical approaches. Here, we briefly discuss the structure of organisms from a complex systems perspective and how much we do and don’t know about their underlying networks. There are obviously a daunting variety of organisms and many levels of organization within them; we try to stay at a general level to enunciate principles that will apply to studies of any biological systems at the organism level or lower that are composed of networks, whether they be biochemical networks within cells or networks of tissues or brain regions. We then argue that, despite an imperfect knowledge of the finest-scale details, there is a coherence to biological states that suggests that we should be able to measure organismal state even with highly imperfect knowledge of the underlying networks. We use this framework to review a number of methods to infer organismal state via sparse sampling of networks, and to suggest future avenues for further development of such methods. Our previous research in aging leads us to use examples primarily from this field, but the conclusions are much more general.

## An Organism as a Complex Dynamic System

There can be little doubt that organisms are complex dynamic systems, as they exhibit all the hallmarks of such systems: they are composed of multiple elements which interact with feedback (and also feedforward) mechanisms; the elements are organized both hierarchically and modularly; and there are clear emergent properties at the various hierarchical levels which emerge from the underlying dynamics at adjacent levels (e.g., Podobnik et al., 2017, 2020). These properties are also shared by other key examples of complex dynamic systems: economies, ecosystems, weather systems, societies, traffic systems, etc. However, organisms—and, more broadly, sub-organismal biological systems—exhibit some unique features that are not shared by all complex dynamic systems:

1. **They are highly optimized via natural selection** (Kriete, 2013). While systems such as economies or ecosystems may undergo weak selective pressure that affects their evolution, biological systems have had billions of years to become fine-tuned, with progress made in each generation conserved and transmitted via the genetic code. Molecular signaling pathways, for example, are much more fine-tuned than economic regulatory policy.
2. **Accordingly, they are goal-directed**. Weather systems, ecosystems, and most other complex dynamic systems simply exist, without an effort to achieve anything. Biological systems, however, have been fine-tuned for a reason: to maximize organismal fitness (roughly speaking). And in turn, for most of the biological machinery, this equates to optimizing physiological/biological equilibrium in the face of constant internal and environmental variation (Cohen et al., 2012; Podobnik et al., 2017, 2020), as denoted by concepts such as homeostasis, homeodynamics, robustness, and resilience (Ives, 1995; Sterling, 2015; Ukraintseva et al., 2016). In other words, biological systems are *designed* (though not in a theological sense) to maintain organismal balance.
3. **Biologically relevant information is conserved over long timescales**. The genetic code permits the conservation of information across very long timescales—billions of years. Many other complex dynamic systems do not really conserve information at all, and those that do are either much more recent (e.g., writing in economic systems, code in computer systems), and/or the information is much less precisely transmitted (cultural transmission).
4. **Accordingly, biological systems are perhaps the most complex systems that exist**. The combination of natural selection, very long timescales, goal-directedness, and information conservation has permitted biological systems to become what is likely the most structured complex system known. New levels and layers of biological organization are constantly being discovered (see below).

It is this last point, perhaps, that explains why biologists are so far behind other fields in adapting complex systems thinking. There was simply too much information, and too much structure, for classic complex systems methodologies to be broadly useful. The advent of -omics and imaging technologies and big-data capabilities are changing this now, but the challenge remains that our basic knowledge of biological organization is still rudimentary.

## What We Do and Don’t Know About Biological Organization

At least since the discovery of the structure of DNA, our knowledge of biological organization has been increasing rapidly. As this process has unfolded, there has been a repeated theme: scientists at each stage fail to appreciate how much more detail has yet to be uncovered. For example, new hormones—key molecules for regulatory coordination—are regularly discovered, such as hepcidin (Nemeth et al., 2003) and apelin (Vinel et al., 2018). Perhaps the best example is the changes that have occurred to biochemistry’s “central dogma” over the years. Once it was established that DNA is transcribed into messenger RNA (mRNA), and that mRNA is translated into proteins, this transfer of information (genes—> mRNA—> protein) became known as the “central dogma” (Crick, 1970). Proteins were considered the key biological effectors. However, it was not long before this dogma began to break down at a number of levels: reverse transcriptases from viruses such as HIV can reverse the flow of information (Spiegelman et al., 1971). Perhaps more importantly, the central dogma radically underestimates the sophistication of the information processing. A single gene can be spliced into multiple mRNAs (Chow et al., 1979). Many new types of RNAs, such as micro RNAs and snoRNAs, are being discovered, often with key signaling roles (Scott and Ono, 2011).

Here, we will look in detail at the recent discovery of “alternative proteins” (Brunet et al., 2020), a telling example of how new layers of biological organization are constantly being uncovered, and how each time they are, our understanding as relates to the application of complex systems methods to biology would need to be re-thought. Since the discovery of alternative mRNA splicing in eukaryotes– i.e., that a single gene could produce many mRNA isoforms—part of the revised central dogma, implicit or explicit, has been that a mature mRNA (i.e., one that has already undergone all splicing and processing before being translated into a protein) codes for one and only one protein. A few exceptions had been detected, but they were considered just that: exceptions. However, with the advent of high-throughput technologies, it was recognized in the early 2010s that most mature mRNAs contained multiple start codons, the series of three nucleotides in the mRNA that signals the ribosome to start translation (Ingolia et al., 2011; Vanderperre et al., 2013). As a result, it was possible that multiple proteins were being translated from a single eukaryotic mRNA. By combining the presence of start and stop codons in the mRNA sequences, it was predicted that the average mature mRNA might code for 7.8 proteins, with some mature mRNAs coding for as many as 89 (Open Prot database v1.6: Brunet et al., 2019), leading to the possibility that the proteome was many-fold larger than previously thought. Indeed, subsequent experiments have shown that many of the so-called “alternative proteins”—proteins that are coded by a mature mRNA, other than the “reference protein” that was canonically expected—are indeed produced by cells. They have been detected by mass spectrometry and by ribosomal profiling, are often highly conserved evolutionarily, and in some cases they have been shown to have crucial roles in biological processes and diseases, often working in tandem with other proteins produced by the same mRNA (Samandi et al., 2017; Chen et al., 2020). Hence, in contrast to the general belief that polycistronic mRNAs are restricted to prokaryotes, an ever-increasing fraction of mRNAs in eukaryotes are known to encode at least two different proteins.

Not all alternative proteins predicted by mRNA sequences have been shown to be translated or to have important biological roles, and there is currently substantial uncertainty as to how many of the alternative proteins will prove biologically important, but the number would certainly appear to be in the thousands, if not much higher. Indeed, the line between “biologically important” and “translational noise” may not be so clear: many alternative proteins likely arise via mutations generating random start codons, but the proteins so-generated may have some biological activity. If that activity is strongly harmful, natural selection should quickly eliminate the mutation, but if the novel protein, due to biochemical stochasticity, is anywhere close to neutral, there may exist a long period where it is produced in small quantities and subject to gradual selection toward a beneficial role, even if it does not play a core regulatory function. There is thus substantial potential for alternative proteins to play broad but hard-to-pinpoint regulatory roles, even beyond the substantial number that are in the process of being identified as key players.

**FIGURE 1:**
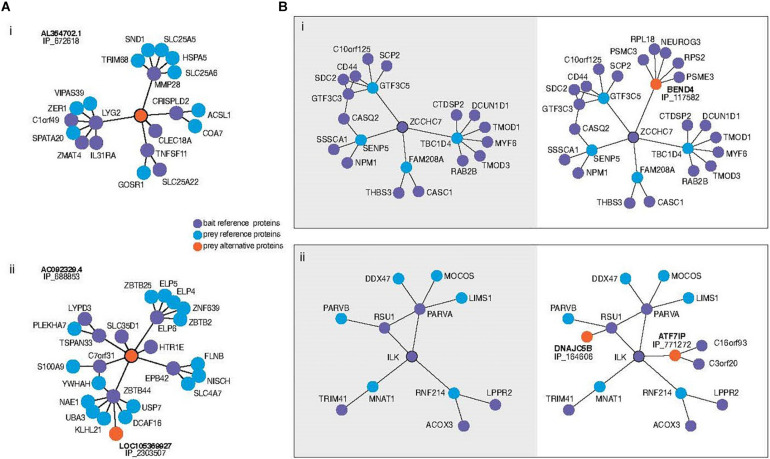
**(A)** Subnetworks of alternative proteins identified in the most comprehensive human protein-protein interaction network (Leblanc et al., 2020). Direct neighbors and neighbors of neighbors (here called second neighborhood) are shown around alternative proteins encoded by genes of “non-coding” biotypes revealing previously unknown physical interaction network structure. Alternative protein IP_672618 relates regions otherwise not connected (i). The authors speculate that the bridging role of IP_688853 between ELP6 and other proteins could yield insight into the mechanism of tumorigenesis recently associated with this gene. Bait proteins are reference (annotated) proteins expressed with a tag for purification from which prey proteins are identified. “IP_” protein accessions refer to OpenProt 1.6 unique identifiers for alternative proteins. **(B)** Second neighborhoods of two reference proteins extracted from the same network with (right) and without (left) alternative proteins. Inclusion of an alternative protein from the dual coding gene BEND4 reveals the addition of a hub around the protein ZCCHC7 (i). Addition of two alternative proteins in the second neighborhood of ILK increases the betweenness centrality of the ILK-RSU1-PARVA clique (ii).

Among those alternative proteins that are emerging as having clear functions, recent work is permitting us to understand how they integrate into biological networks. Briefly, data generated by high throughput mass spectrometry (MS) experiments have been re-analyzed with the inclusion of alternative proteins in the library used at the spectra-peptides matching step (Leblanc et al., 2020). When these data result from separate purification of multiple tagged proteins followed by MS analysis it is possible to confidently identify alternative proteins in a network of protein physical interactions. Interestingly, a surprising number appear to have roles linking what otherwise appear to be separate modules, or as hubs (Figure 1). In other words, the structure of the regulatory network, including important higher-order network properties, could completely change with or without the inclusion of the alternative proteins. Indeed, the addition of alternative protein IP_688845 in the interactome of the ELP6 protein may yield insight surrounding its recent association with tumorigenesis and migration of melanoma cells (Close et al., 2012; Figure 1Aii). The re-analysis shows that the novel interactor bridges this member of the elongation complex to protein clusters which include S100A9, recently identified as biomarker up-regulated in metastatic melanomas (Wagner et al., 2019) and TSPAN33, a protein which modulates cell adhesion and migration through its effect on plasma membrane mechanical properties (Navarro-Hernandez et al., 2020). Several hypotheses could be explored with this larger subnetwork as a starting point. While it is not yet proven whether these changes in our understanding of network structure are expected to lead to changes in the predicted functional dynamics, there is every reason to expect that this would be the case. In similarly generated data, alternative proteins have even been observed in the interaction network of viral proteins in Zika virus infected human cells (Leblanc and Brunet, 2020).

The changes in biological paradigm implied by the existence of a broad range of functional or semi-functional alternative proteins should have been expected. Why would occasional mutations not have produced additional start codons? Why would those additional proteins not have been translated? And why would natural selection not have then subsequently acted on them, leading in all likelihood to the sharing of a mature mRNA as a way to improve coding efficiency of the genome and coordination of related regulatory functions? We would argue that this example is a clear microcosm of how the combination of stochasticity, biological information conservation, and billions of years of natural selection has added multiple layers to the structure of biological organization.

We have clearly not reached the bottom yet, and even once (if?) we do identify all the molecular players, a full functional mapping of their interactions, including the weak non-primary relationships, is certainly still well in the future. Moreover, network structure itself may not be a fixed property of an organism. Recent developments have shown that the structure of protein-protein interaction (PPI) networks correlates with cell type (Huttlin et al., 2020). In this study, the authors of the BioPlex network have constructed PPI networks of two different cell types using the method described above and observed largely the same proteins engaged in different interactions. In other words, rewiring of PPI networks correlated with system state, adding another major challenge and level of complexity in our ability to infer biological network structure.

In turn, this lack of ability to fully map biochemical networks could be an important barrier for our ability to apply key tools from complex systems theory—notably network theory/graph theory—to these networks. Of course, we do not mean to imply that network theory has no applications in biology. Indeed, there will be clear applications in the simplest systems (Nijhout et al., 2017), or when emergent phenomena are clear enough to create higher-order networks that can be mapped (Bashan et al., 2012). But while the ability to predict and control nematode behavior through a network of 40 neurons is on the one hand an excellent and impressive demonstration of the power of network theory (Yan et al., 2017), it is also a humbling demonstration of how much more would be required to achieve similar prediction/control of human behavior, particularly when the number of neurons and their connection structure is not fixed from one individual to another.

In short, we argue that most models of biological networks are—and, for the foreseeable future, are likely to remain—*sparsely sampled systems*, systems in which the nodes and their edges are insufficiently known or sampled to robustly and directly characterize network-level properties. In such systems, insights will need to come not through exhaustive data, but through judicious use of methods to extract the relevant signals despite the sparseness of the data. This is true despite the advent of massive -omics, and despite the data processing tools that are being developed to manage that information.

## Data Tools for Sparsely Sampled Networks

While the above situation might sound pessimistic with regard to our potential to make progress in understanding biology, it is anything but. In fact, the same system properties that make biology so complex and hard to map also make it potentially more tractable as a complex system with the right analytical tools. It is the goal-directedness of biological systems that gives their components teleological functions: hearts are for pumping blood through the circulatory system, B-cells are for generating the antibodies in the adaptive immune response, and ribosomes are for translating mRNA into proteins (teleological is used here in the sense that purposeful behavior of systems can arise from purposeless evolution via natural selection, a shorthand that allows reduction of otherwise intractable, purely descriptive, mechanistic models). There is no equivalent set of functions in, say, an ecological network: what are flower-pollinator relationships, or plankton communities in marine ecosystems, “for”? And indeed, rightly or wrongly, for centuries biologists have relied on this kind of teleological understanding of biological components without a second thought.

**FIGURE 2:**
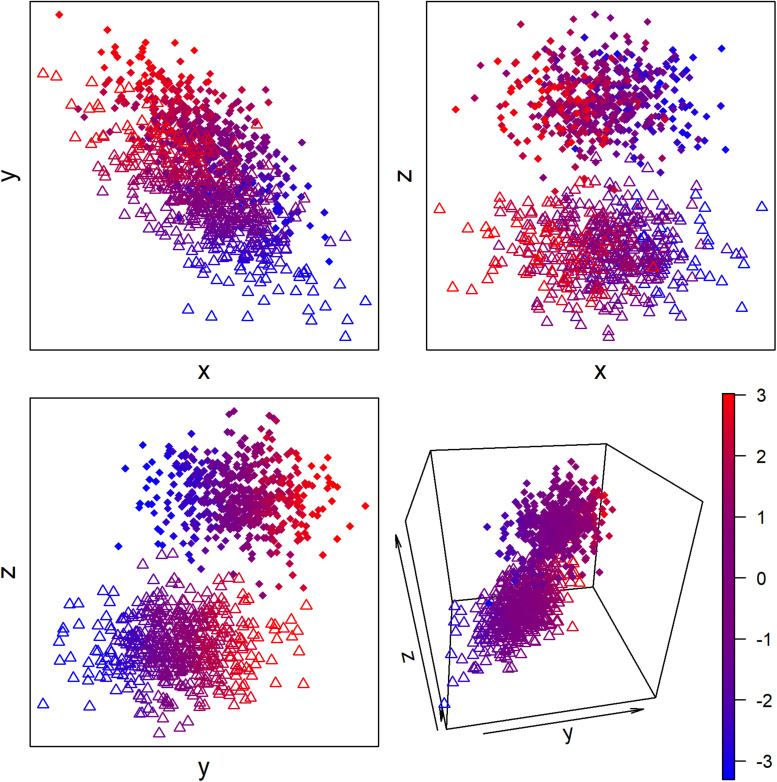
Hypothetical example of continuous and discrete latent biological states measured with three proxy biomarkers. Color represents the latent continuous state, and circles vs. triangles represent the discrete state. The proxy biomarkers x and y but not z are associated with the continuous state, and the proxy biomarkers y and z but not x are associated with the discrete state.

More generally, we can conceive of most biological regulation as adjusting the condition of the organism to maintain broad-sense homeostasis. This regulation can occur across two types of dimension: discrete and continuous (Figure 2). For example, migratory birds arriving for the breeding season go through a discrete transition to a breeding state (Jacobs and Wingfield, 2000; Williams, 2012). This involves not just changes to sex hormones and gonad size, but also changes in immune function, diet, metabolism, and likely a host of other aspects of physiology that have not been studied extensively. We can thus conceive of breeding vs. non-breeding state as two “attractor states” of the system, in complex systems terminology. Small changes in physiology within a state are generally not sufficient to make the organism jump from one “attractor basin” to the other—only the right, and sufficient, stimulus can affect the change. Other discrete states could include sleeping vs. wakefulness, an immune system fighting acute infection, or the dauer stage of *C. elegans*.

Continuous variation occurs along a gradient. The chronic low-grade inflammatory response characteristic of mammalian aging is a good example (Franceschi et al., 2000), as is hunger, or the metabolic changes that accompany continuously increasing levels of exercise intensity. Both continuous and discrete processes are reflected in multiple, connected aspects of biology. Biological changes rarely happen in a vacuum, but involve coordination across multiple components that need to take into account what other parts of the system are doing (Csete and Doyle, 2004). And this is the property of biological systems that opens them up to tractability: the *coherence* of the overall system state. A biological system is, at any moment, in a state that reflects all the discrete and continuous dimensions of which it is composed: for example, a bird might be breeding, experiencing an acute infection of type X, under conditions of abundant food, while resting, etc. We do not yet have a complete enumeration of all these dimensions for any organism, and perhaps not even for any biological component. And these dimensions may not be additive: the presence of one state may preclude or modify others, and many of the biochemical components that help determine states are in fact shared. For example, the inflammatory molecule C-reactive protein (CRP) is involved in both acute infections, and in low-grade chronic inflammation associated with aging. CRP level is thus not a simple measure of either of these processes, but must be integrated with other information to give information about either (Bandeen-Roche et al., 2009; Morrisette-Thomas et al., 2014).

Broadly speaking, we can summarize the situation as follows: we have a set of biomarkers (taken here to mean any indicators of biological state, but likely individual molecules in biological networks), and we would like to make robust inferences about the state of the organism/cell/other biological component (let’s call it an organism for simplicity). On the one hand, we have the threefold challenges of (1) imperfect knowledge of the states the organism might be in, (2) imperfect knowledge of how the biomarkers might interact with each other and/or overlap in how they signal different states; and (3) a marked undersampling of the relevant molecules (we assume). On the other hand, the associations among the biomarkers should reflect the structure of the underlying networks and the covariation in the biomarkers this generates: the coherence of the system state. Below we discuss several statistical approaches that can be used to extract information from such ensembles of biomarkers representing sparsely sampled networks.

### Principal Components Analysis

Principal components analysis (PCA) is a data reduction technique that reorganizes the information in a large set of semi-redundant variables, permitting the user to extract a (much) smaller number of variables that explain the bulk of the variation in the original set. It is related to many other methods, such as factor analysis and t-SNE (Van Der Maaten and Hinton, 2008), that can obtain similar objectives through slightly different approaches. Our discussion here applies equally to all these methods.

While PCA is primarily considered or used as a way to reduce the number of dimensions in a dataset, it is also a powerful tool to understand those dimensions. For example, application of PCA to epidemiological data on malaria in India showed that seven indices could be effectively reduced to one, not two, PC axes (Cohen et al., 2010). This meant that indices showing the balance between the two main malaria species and those showing the abundance were actually moving largely in tandem, implying an ecological gradient dynamic in which one species dominates when abundance is high, and another dominates when abundance is low. Similarly, PCA analysis of inflammatory markers in human cohort data has shown that a single key axis describes much of the variation in a population of largely older adults, and that pro- and anti-inflammatory markers positively co-vary along this axis: chronic inflammation is not characterized by high pro-inflammatory markers and low anti-inflammatory markers, but by high levels of both, an activation of the system in which the anti- markers chase the pro- markers without ever really catching up (Bandeen-Roche et al., 2009; Morrisette-Thomas et al., 2014; Varadhan et al., 2014; Cohen et al., 2018a). Similarly, PCA applied to standard clinical biomarkers has revealed a surprisingly stable structure that integrates multiple physiological systems (Cohen et al., 2015b). The first principal component is multi-systemic, with high scores indicating anemia, low calcium, low protein transport (e.g., albumin), and high chronic inflammation, and was termed “integrated albunemia.” Integrated albunemia scores increase with age and predict mortality risk and clinical frailty but not chronic diseases. They are also elevated acutely during hospitalizations, etc. The correlation structure among the main implicated systems appears stable, such that integrated albunemia is a more robustly measurable phenomenon than its component markers. For example, its correlation with age and disease is more stable than that of the component markers. This appears to be a good example of a continuously varying attractor state.

While PCA, when used appropriately, can be a powerful tool to uncover structure in biological data, it needs to be used with caution. In particular, it can be sensitive to population composition, and structure can also vary depending on conditions or population (Pigeon et al., 2013). For example, application of PCA to 13 circulating biomarkers in wild European starlings (*Sturnus vulgaris*) showed that the correlation structure—and thus the axes one might extract—varied across years and breeding season (Fowler et al., 2018). This is to be expected: changes in food abundance, diet composition, or parasite burden should be expected to vary across seasons and years, and also to cause changes in which biomarkers correlate with which. But if the correlation structure is unstable, how can we extract axes? One answer is that partial correlation structures can be extracted. For example, in bighorn sheep (*Ovis canadensis*), milk composition varies across both years and across individual mothers, and it was possible to extract a correlation structure for each of these aspects using appropriate hierarchical models (Renaud et al., 2019).

However, more generally, care should be used to apply PCA and related methods with appropriate cross-population validation to ensure that any findings are true reflections of biological organization rather than artifacts of sampling, population composition, or environmental heterogeneity (Cohen et al., 2018b). In the case of the Fowler et al. (2018) study, no meaningful axes could be extracted due to the strong changes in correlation structure and the limited samples sizes in the relevant subgroups to robustly estimate structure. In contrast, integrated albunemia has been validated in multiple populations (Cohen et al., 2015b) and even species (Wey et al., 2019). Likewise, there have multiple validations of inflamm-aging as a continuous process that can be identified by PCA and related methods (Bandeen-Roche et al., 2009; Cohen et al., 2015b, 2018a). The simplest test is to repeat the PCA in distinct populations or population subsets, extract the loadings, and then cross-apply them to the other populations to generate scores. This generates multiple versions of the PCA scores as calibrated based on, say, men, women, population 1, population 2, etc. A correlation matrix or correlogram can then be used to assess how well the same axis is extracted. In the case of integrated albunemia, for example, these correlations are generally greater than 0.95, even from populations on different continents. Further confirmation can be obtained by ensuring that the interpretation of the axis via its loadings is similar. Graphical approaches to this can be found in Cohen et al. (2015b).

### Statistical Distance

Statistical distances are ways of quantifying how different an individual or group is from another individual or group, usually across a series of variables. In the context of measuring biological or physiological state, the principal application has been to quantify how different an individual’s multivariate biomarker state is from some reference state, often the population average or some reference healthy state. This requires statistical distances that can quantify a distance between an individual and a group. The two main methods that do this are Euclidean distance and Mahalanobis distance (De Maesschalck et al., 2000). Euclidean distance does not take into account any correlations between the biomarkers, and thus, if redundant markers are included, will double-count that information. Mahalanobis distance assumes multivariate normality, and then uses the inverse of the correlation matrix to eliminate redundancies among highly correlated variables. This has the effect of down-weighting redundant variables.

Mahalanobis distance has, accordingly, been more widely used to measure physiological state. To our knowledge, the first application was to measure physiological declines prior to death in fruit flies (*Drosophila melanogaster*) (Shahrestani et al., 2012). It was subsequently and independently applied to human biomarker data for similar purposes, and has also been applied in wild animals, both with (Milot et al., 2014) and without (Fowler et al., 2018) success. The assumption in all these cases is that average state is close to optimal state, and that individuals far from optimal are likely to be more unhealthy. This assumption is related to theory suggesting that homeostatic states are relatively homogeneous, whereas there are numerous ways that homeostasis can be lost and thus a diversity of ways to diverge from the norm (Cohen, 2016). Accordingly, Mahalanobis distance has been proposed as a measure of homeostatic or physiological dysregulation. This proposition makes a number of predictions. First, Mahalanobis distance should increase with age. Second, it should predict a wide variety of adverse health outcomes after controlling for age. Third, this signal should not depend strongly on any single biomarker, implying that (a) the choice of biomarkers to include is not crucial, and (b) the signal should increase monotonically (but with diminishing marginal returns) as the number of biomarkers is increased. All of these predictions have now been confirmed by multiple studies (Cohen et al., 2013, 2014, 2015a, 2018c; Arbeev et al., 2016; Dansereau et al., 2019; Kraft et al., 2020; Liu, 2020). The third prediction is particularly important from a complex systems perspective, as it implies that the signal is diffuse or distributed in the physiological networks, and thus robustly estimable from subsamples of biomarkers even without detailed knowledge of network structure. It does thus appear that Mahalanobis distance is a valid metric of homeostatic dysregulation, though it certainly involves substantial measurement error. However, an advantage of the Mahalanobis distance approach is that it makes no prior assumptions about “good” or “bad” levels of biomarkers, and does not calibrate based on age or anything else (unless a specific reference population is chosen). This makes it agnostic and neutral for subsequent physiological inferences.

Mahalanobis distance can also be applied to particular physiological systems by dividing biomarkers into groups based on *a priori* knowledge. Indeed, *a priori* knowledge does a reasonably good job of distinguishing systems with minimally correlated dysregulation levels (Li et al., 2015). Net of age, correlations among physiological systems measurable with standard clinical human biomarkers are generally significant but weak (*r* < 0.2), implying feedback effects among the systems. This led to the prediction that similar types of systems might be identified in -omics data, notably DNA methylation, gene expression, or proteomics. However, this has not yet been proven, and indeed in gene expression data from human blood samples, the opposite pattern emerged: most systems identified via gene ontology did not show any significant correlations with age, and those that did show correlations that replicated across datasets were uniformly negative rather than positive (Dufour et al., 2020). This might be indicative of these systems losing responsiveness to stimuli with age, but that remains speculation at this point.

### Cluster Analysis

While PCA and Mahalanobis distance are both good measures of continuous processes, cluster analysis is more appropriate for detecting discrete states. Like PCA and Mahalanobis distance, many clustering algorithms are largely agnostic/uncalibrated/unsupervised, generating clusters based on similarities or differences in the data rather than an external target. There are many clustering algorithms, each with strengths and weaknesses, and a review is beyond the scope of this article (Scheibler and Schneider, 1985; Budayan et al., 2009). Hierarchical clustering, for example, has been profitably applied to biomarker data (Sebastiani et al., 2017). In some sense, clustering can be thought of as a discretized version of PCA, for when the target process states are discrete rather than continuous. A challenge here is that it is not always possible to know whether the states/processes of interest are continuous or discrete *a priori*. In fact, in some cases, the set of biomarkers in question may capture both discrete and continuous processes (Figure 2). When there is doubt, it may be advisable to try both methods, with the objective of assessing the discreteness of the phenomena in question. Because of measurement error, biologically discrete processes may in fact appear overlapping, so the criterion for use of cluster analysis should be the presence of clear, though potentially overlapping, aggregates in multidimensional space. Fuzzy clustering methods may be appropriate in such cases (Budayan et al., 2009). Also, given that biological networks are both weighted and directed, it may be relevant to apply clustering methods specifically designed for such networks (Clemente and Grassi, 2018; Barajas-Martínez et al., 2020).

A second challenge with cluster analysis is the diversity of methods available. These methods often give discordant results, identifying clusters that are not necessarily similar in their composition from one method to the next. This is, to some extent, to be expected. If we choose a group of 100 people and try to cluster them based on similarity, our groups could look very different if we base the similarity on demographics, on health state, on music preferences, etc. There is no “true” way to cluster the people. Even with the same set of variables, the way they are weighted and treated should be expected to have an impact. Nonetheless, if the clusters reflect true biologically discrete states, we should expect a certain reproducibility across methods. In this sense, cross-method validation of cluster analysis could prove an important tool for identifying important biological attractor states.

### Statistical Network Inference

It is possible to combine analysis of correlation structure and clustering methods to infer network structure. This has been done both with general physiological biomarkers (Barajas-Martínez et al., 2020), and with -omics data, for example using the weighted gene coexpression network analysis (WGCNA, Zhang and Horvath, 2005; Hariharan et al., 2014). The idea here is that the correlation structure of variables reveals the network structure, and in the context of high-dimensional -omics data may also reveal discrete modules. We view this approach as one with great potential, but also one that needs to be used with caution. In particular, substantial work needs to be done to assess the robustness of the estimation of network structure to data stochasticity. The Cohen lab has unpublished analyses on gene expression data in which WGCNA failed to produce even minimally similar structures across different datasets; this may be due to the difficulty correcting for batch effects and how this impacts correlation structure, or to population differences in correlation structure (Dufour et al., 2020). It is well-known that correlation structures do change, across age (Barajas-Martínez et al., 2020), across environment (Fowler et al., 2018), and across cell type (Huttlin et al., 2020), among others. Such instability of the basic network structure could be problematic, but also could be the feature of interest (Fowler et al., 2018; Barajas-Martínez et al., 2020). It is also worth noting that such methods generally characterize the network structure of a population, but do not permit direct evaluation of an individual’s physiological state. If network structure is itself malleable, network structure may simply be one more indicator of the attractor state of the individual, in which case methods that quantify individual state could serve as a proxy for network structure after appropriate validation. This could open up many potential research directions.

### Machine Learning

PCA, Mahalanobis distance, and cluster analysis are relatively neutral methods that make few assumptions about the data. They do need to be used in biologically informed ways—for example, choice of variables and appropriate cross-population validation for PCA, and choice of reference population for Mahalanobis distance—but beyond this they are largely agnostic, or, in machine learning terms, unsupervised. In contrast, there are a host of supervised machine learning methods that have arisen in the last decades that permit the generation of precise algorithms to predict targets. For example, chronological age or measures of health status have been used to train random forests and deep neural nets applied to clinical biomarkers (Putin et al., 2016; Bello and Dumancas, 2017), and elastic nets or other regression-linked approaches applied to DNA methylation data and metabonomics data (Horvath, 2013; Hertel et al., 2016). However, such methods present substantial challenges when there is not a clear biological framework to link the biomarkers to the prediction target (Hertel et al., 2019), a symptom of a much more general challenge in artificial intelligence (Mitchell, 2020). These techniques are thus undoubtedly powerful, but work best when the biological nature of the prediction target is crystal clear, and are thus of limited interest in the context of this review. That said, there are many applications of the newer machine learning methodologies that can be applied in an unsupervised way for the characterization of complex biological systems. They are too numerous to enumerate here, as our objective is less an exhaustive review than a demonstration of principle which we hope our readers will take in new directions.

## Discussion

We have argued here that a complex systems perspective on biological structure leads to reasons for both pessimism and optimism in terms of our ability to measure important biological states. On the pessimistic side, we are a long way from even identifying all the key molecules in the relevant biological networks, much less a full map of their pairwise interactions. Furthermore, the complexity of the underlying networks (and experience) both indicate that single biomarkers will often be generally poor indicators of system state (Cohen et al., 2018b). This is partly because the underlying networks are generally structured to adjust multiple outputs as a function of multiple inputs, requiring a regulatory network structure that involves multiple intermediary pathways that balance the potentially competing signals of the inputs, much as in a neural network or autoencoder (Csete and Doyle, 2004; Cohen et al., 2020). Because of this, it is rare that a single molecule has a universal interpretation. For example, interleukin-6 (IL-6) is widely considered the best marker of chronic inflammation. However, there are different types of inflammation, and IL-6 can even have anti-inflammatory roles in some contexts (Bandeen-Roche et al., 2009). It is a decent marker on average, but performs worse than multivariate approaches designed to integrate across numerous inflammatory cytokines (Cohen et al., 2018a).

On the optimistic side, the basic coherence of biological function implies that key biological states should be measurable with small ensembles of molecules, often arbitrarily chosen within the sphere of general interest. Full knowledge of the network is not needed to make progress. This assertion bears some qualitative similarities to the field of compressed sensing (Donoho, 2006), though we note that in that case there is an assumption of “sparseness” in a technical sense (i.e., most coefficients are zero), whereas we have referred to sparseness not of coefficients, but of data, and in a more colloquial sense. We thus do not believe, though cannot yet prove, that there is no formal link between our assertions here and compressed sensing.

Between the optimism and the pessimism lies the notion of biological function, the teleological glue that holds together most of our understanding of biology. Physical structures (e.g., tissue organization) and network structures (e.g., biochemical pathways) are thought to have functions, in some cases patently obvious, in some cases discovered through careful research, but in many cases still obscure. The approaches outlined here are examples of ways to elucidate network structures that may have gone unremarked, and in particular to link such structures to functions. For example, if cluster analysis of single-cell gene expression reveals three stable yet heretofore unremarked profiles of what was previously thought to be a homogeneous cell type, there would be good reason to conduct further research on these three types in order to elucidate their respective roles.

In some sense, this assertion is completely unremarkable—of course we would explore the functions of such cell types if we found good evidence of distinctions, and cluster analysis is hardly new—and yet it also opens the door to a new framework for how we should explore biological variation. To date, we have tried to fit biological variation into our notions of how biology is organized, with varying degrees of success. For example, as noted above, many machine learning approaches have been applied to various kinds of data in an effort to quantify the aging process, without questioning the assumption that there is an aging process that could be measured. However, the existence of “aging” as a biologically (as opposed to culturally) meaningful concept is now being questioned (Cohen et al., 2020). Occasionally when efforts to map data onto existing concepts fails, a new paradigm emerges from the data, such as the alternative proteins discussed above. But the judicious use of methods such as those described here offers the potential for much more: a systematic and data-driven exploration of the structure, and thus function, of biological organization.

In short, we are proposing a broad effort to map biological variability based on several principles:

(1) **Analyses should be largely data-driven and conceptually agnostic.** We often do not understand the key structures, states, and processes, and these may or may not map well to the concepts and words we employ to describe them. However, general biological knowledge should be used to structure the analyses. Note that the annotations underlying the data are reflections of our biological conceptions and assumptions (Brunet et al., 2018), so there is no perfectly agnostic approach.
(2) **Replicable patterns in the data indicate key aspects of biological organization.**
(3) **Identifying structures and patterns will point to function** (Han, 2008). And function, once determined, will give meaning to the findings.
(4) **Collect information on context**. For example, information on an organism’s sex, age, environment, etc. could prove essential to uncovering the functional relevance of different states.

Note that the approach we propose also avoids one of the primary criticisms possible for a teleological or functional (as opposed to mechanistic) understanding of biology: that our conceptions of function are biased by cultural or other subjective factors. In fact, a data-driven approach to identifying functional biological units could help actively identify cases where subjective factors have unduly influenced our understanding, while simultaneously permitting us to harness the explanatory power of natural selection as a force that shapes networks to achieve certain functional objectives.

The methods illustrated above—PCA, statistical distance, and cluster analysis—are examples, not recommendations: many others could be employed, and new ones will be developed, particularly as we move toward integration of multi-omics. The key point is that even standard, well-known techniques can reveal hidden structures in biological data, allowing the data to tell us a story that is often quite different from what we expected. For a long time, biological research has been largely hypothesis driven, an approach that works well with smaller data scales and when every variable is carefully chosen and laboriously measured. But in the world of big data and -omics, we are faced with the scale of our ignorance and the impossibility of generating enough well-founded hypotheses. The approach we propose here offers a way forward, a basic mapping of the landscape of biological function through networks, on which future hypotheses can be built.

## Author Contributions

AC conceived of and wrote the main manuscript. SL created Figure 1 and wrote sections on alternative proteins in consultation with XR. All authors revised and edited the full manuscript.

## Conflict of Interest

AC was founder and CSO at Oken Health. The remaining authors declare that the research was conducted in the absence of any commercial or financial relationships that could be construed as a potential conflict of interest.
